# Semi-Automated Quantification of Retinal and Choroidal Biomarkers in Retinal Vascular Diseases: Agreement of Spectral-Domain Optical Coherence Tomography with and without Enhanced Depth Imaging Mode

**DOI:** 10.3390/diagnostics12020333

**Published:** 2022-01-27

**Authors:** Lucy J. Kessler, Dmitrii Bagautdinov, Grzegorz Łabuz, Gerd U. Auffarth, Ramin Khoramnia

**Affiliations:** 1 Department of Ophthalmology, University of Heidelberg, 69120 Heidelberg, Germany; LucyJoanne.Kessler@med.uni-heidelberg.de (L.J.K.); dmitrii.bagautdinov@med.uni-heidelberg.de (D.B.); Grzegorz.Labuz@med.uni-heidelberg.de (G.Ł.); Gerd.Auffarth@med.uni-heidelberg.de (G.U.A.); 2 HEiKA—Heidelberg Karlsruhe Strategic Partnership, Heidelberg University, 69120 Heidelberg, Germany; 3 HEiKA—Heidelberg Karlsruhe Strategic Partnership, Karlsruhe Institute of Technology (KIT), 76131 Karlsruhe, Germany

**Keywords:** optical coherence tomography angiography, biomarkers, enhanced depth imaging, automated quantification, hyperreflective foci, choroidal vascularity index, ellipsoid zone reflectivity ratio, retinal vascular diseases

## Abstract

Background: We compared with and without enhanced depth imaging mode (EDI) in semi-automated quantification of retinal and choroidal biomarkers in optical coherence tomography (OCT) in patients with diabetic retinopathy (DR) or retinal vein occlusion (RVO) complicated by macular edema. We chose to study three OCT biomarkers: the numbers of hyperreflective foci (HF), the ellipsoid zone reflectivity ratio (EZR) and the choroidal vascularity index (CVI), all known to be correlated with visual acuity changes or treatment outcomes. Methods: In a single examination, one eye of each patient (*n* = 60; diabetic retinopathy: *n* = 27, retinal vein occlusion: *n* = 33) underwent macular 870 nm spectral domain-OCT (SD-OCT) B-scans without and with EDI mode. Semi-automated quantification of HF, EZR and CVI was applied according to preexisting published protocols. Paired Student’s *t*-test or Wilcoxon rank-sum test was used to test for differences in subgroups. Intraclass correlation coefficient (ICC) and Bland–Altman plots were applied to describe the agreement between quantification in EDI and conventional OCT mode. The effect of macular edema on semi-automated quantification was evaluated. Results: For the entire cohort, quantification of all three biomarkers was not significantly different in SD-OCT scans with and without EDI mode (*p* > 0.05). ICC was 0.78, 0.90 and 0.80 for HF, EZR and CVI. The presence of macular edema led to significant differences in the quantification of hyperreflective foci (without EDI: 80.00 ± 33.70, with EDI: 92.08 ± 38.11; mean difference: 12.09, *p* = 0.03), but not in the quantification of EZR and CVI (*p* > 0.05). Conclusion: Quantification of EZR and CVI was comparable whether or not EDI mode was used. In conclusion, both retinal and choroidal biomarkers can be quantified from one single 870 nm SD-OCT EDI image.

## 1. Introduction

Optical coherence tomography (OCT) has gained a pivotal role in the diagnosis and therapy surveillance of retinal diseases such as diabetic retinopathy or retinal vein occlusions complicated by macular edema that requires anti-vascular endothelial growth factor (VEGF) or corticosteroid therapy. OCT is a safe, non-invasive and fast technique for the visualization of the retinal structures [1]. The treatment is essentially guided by the changes of retinal layer thickness variation, and this can be quantified by OCT imaging [2,3]; however, individual treatment responses are not well understood, and it remains challenging to predict visual outcomes. With a spatial resolution of up to 8–10 µm/pixel in the axial direction, OCT facilitates the detection of small pathological structures or alterations in the retinal layers such as hyperreflective foci or ellipsoid zone interruptions [4]. These morphological alterations can act as biomarkers and may provide further information about the stage of the disease or the potential treatment response. There are reports that among these emerging OCT biomarkers, some can be associated with the visual outcome in various retinal diseases: hyperreflective foci (HF), ellipsoid zone reflectivity ratio (EZR) and choroidal vascularity index (CVI) [5,6,7,8,9,10,11].

Hyperreflective foci in OCT are small (<30 µm), solitary hyperreflective dots without shadowing. Several authors proposed that HF may be activated microglia cells, thus representing the grade of local inflammation. An association has been observed between hyperreflective foci and poorer visual outcomes in diabetic macular edema and retinal vein occlusion [5,6]. The ellipsoid zone is thought to represent the inner segments of the photoreceptors and its hyperreflectivity is assumed due to high mitochondrial density, thus indicating the vitality of photoreceptors [12]. Previous studies demonstrated a correlation in retinal diseases between EZ recovery and visual function [13,14]. Choroidal vascularity index (CVI) as an OCT biomarker was first described by Agrawal et al., who discovered CVI changes in eyes with uveitis [15]. This index describes the percentage of the vascular area of the total choroidal area in an OCT-B-scan. Dysfunction of the choroid has been observed in diabetic macular edema as well as retinal vein occlusions [9,16,17,18,19].

As OCT technology advanced, different imaging modalities are available that can be applied to focus on certain retinal layers to allow maximal resolution. The enhanced depth imaging mode (EDI) is a feature that is integrated into most SD-OCT devices, and it is supposed to optimize the visualization of the choroid by moving the choroid closer to the zero-delay line [20,21]. Hence, in most reports, choroidal biomarkers have usually been derived from SD-OCT with EDI mode or swept-source OCT (SS-OCT), which allows deeper penetration through the retinal pigment epithelium due to longer central wavelength (~1060 nm compared to ~800 nm in SD-OCT) [22]. In contrast, most biomarkers in retinal layers were generally derived from conventional SD-OCT. In the past, choroidal biomarkers were mostly applied in uveitis or choroidal disorders; however, with the emerging evidence that CVI may be a significant parameter for retinal vascular diseases as well, it would be beneficial to analyze both retinal and choroidal biomarkers in one single image instead of two different OCT scans [8,10,23]. This would save time, reduce the diagnostic burden for the patient and mitigate potential artifacts induced by taking two distinct OCT images at two different time points. Our aim was to compare automated quantification of retinal and choroidal OCT biomarkers in SD-OCT scans with and without EDI mode to conclude whether comprehensive analysis of OCT biomarkers across all retinal and choroidal layers is feasible using one single SD-OCT image in EDI mode.

## 2. Materials and Methods

This was a cross-sectional study conducted at the University Eye Hospital of Heidelberg, Germany. The study was approved by the Research Ethics Committee. All study protocols adhered to the tenets of the Declaration of Helsinki. This study was registered on the German Clinical Trial Register (registration number: DRKS00024399).

### 2.1. Study Cohort

We enrolled 60 patients at our hospital, all on anti-VEGF treatment: 27 with diabetic retinopathy and 33 patients with retinal vein occlusion complicated by macular edema at treatment initiation, and they were followed between September and November 2021. It is noteworthy that, at the time of the cross-sectional study, not all presented with a macular edema; however, the presence of macular edema at anti-VEGF treatment initiation was part of the inclusion criteria. All subjects underwent a comprehensive ophthalmic examination, which included measurement of the best-corrected visual acuity (BCVA), measurement of the intraocular pressure, slit-lamp biomicroscopy, indirect funduscopy and SD-OCT scans with and without enhanced depth imaging (EDI) (Spectralis, HeyEx software version 6.3.2.0, Heidelberg Engineering GmbH, Heidelberg, Germany). Exclusion criteria included age younger than 18 years, retinal or glaucoma surgery in the history, amblyopia, uveitis and presence of other retinal diseases associated with macular edema such as neovascular age-related macular degeneration. Patients with a refractive error of more than six diopter spherical equivalents or OCT imaging of low quality (<30 with 35 being the best signal-to-noise ratio) that impaired analysis were excluded.

### 2.2. Optical Coherence Tomography Acquisition and Analysis

#### 2.2.1. Image Acquisition

OCT scans were obtained using 870 nm Spectralis SD-OCT, version 6.13.3.0 (Heidelberg Engineering GmbH, Heidelberg, Germany). Macular cube line scan protocol was used to obtain the image data. OCT scans with EDI mode were acquired directly after the conventional OCT scan using the referencing tool from the proprietary software to ensure matched location at each B-scan. EDI mode is integrated into the proprietary software. B-scans were applied in high-speed mode and each B-scan was averaged with 50 frames. The foveal B-scan was extracted for further analysis. All images had a signal-to-noise ratio >30 (with 35 being the best score).

#### 2.2.2. Image Processing and Analysis

**Figure 1:**
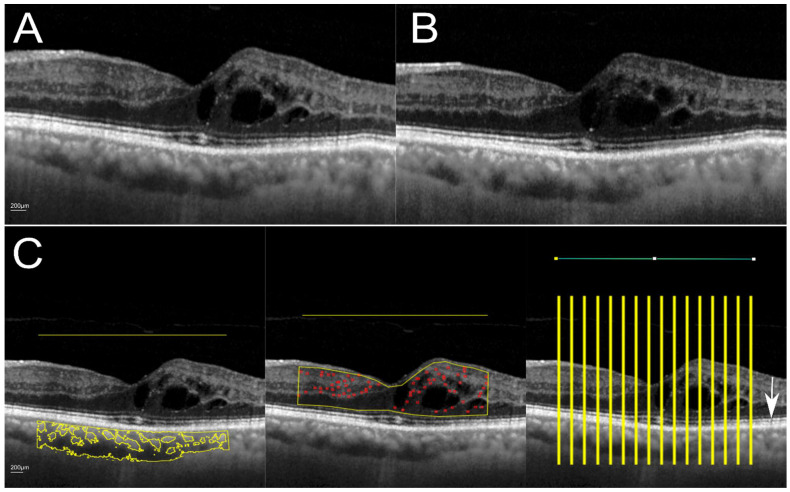
Spectral domain-OCT without (**A**) and with (**B**) enhanced depth imaging mode and (**C**) region of interest according to OCT biomarkers of interest. ROI for CVI and HF was derived from the EDI image. C Left: Choroidal vascularity index was calculated according to the protocol of Agrawal et al. [15]. Middle: For the quantification of hyperreflective foci (red circles), we applied the protocol described by Midena et al. [23] Right: EZR was quantified automatically in 200 µm distances between each ROI (in total 16 measurements). Horizontal line in the upper part of the image represents the central 3 mm area. White scale bar at the bottom left corner in A and C represent 200 µm. White arrow indicates the ellipsoid zone.

Logarithmic-transformed display of OCT was exported as tagged image file format (TIFF) using the integrated proprietary software. Semi-automated quantification of all three OCT biomarkers in the open-source software Fiji (US National Institutes of Health, Bethesda, Rockville, MD, USA. https://imagej.net/software/fiji/ (accessed on 25 January 2022) has been described in the literature [15,23,24,25]. All images underwent a preprocessing of image registration for alignment and histogram-matched normalization before quantification. The region of interest (ROI) was defined as the central 3 mm area. For EZR, we applied the same methods we described in our previous work, where, briefly, a longitudinal reflectance profile was obtained at every 200 µm [11]. Ellipsoid zone reflectivity ratio (EZR) was calculated as the ratio of EZ reflectivity to the retinal pigmented epithelium (RPE) reflectivity, which is considered the most hyperreflective band in OCT. Quantification of hyperreflective foci and the measurement of choroidal vascularity index required a manual selection of the ROI. This mask was outlined in the EDI image and then transferred to the conventional OCT image to ensure that the ROI is the same in both images (Figure 1). For hyperreflective foci, we followed the protocol from Midena et al. [23]. That is, foveal OC B-scan was denoised followed by enhancement of local contrasts and the integrated tool Spot Counter was used for automated quantification of hyperreflective foci in the area between the retinal nerve fiber layer and the external limiting membrane. For choroidal vascularity index (CVI), we followed the protocol from Agrawal et al. [15]. The ROI, which represented the entire choroid thickness in the central 3 mm area, was first defined in the EDI scan. The image was binarized to distinguish luminal and stromal areas within the ROI. By thresholding and composition of selected ROIs, the luminal area within the total choroidal area was measured. The mask of ROI was transferred to the conventional OCT B-scan to conduct the same analysis.

### 2.3. Statistical Analysis

Statistical analysis was performed in IBM SPSS Statistics software, version 27.0 (IBM Corp., Armonk, NY, USA) and Analyse-it statistical software, version 5.10 (Analyse-it Software, Ltd., Leeds, UK). Two-sided *p* < 0.05 was considered statistically significant. Data distribution was tested with Kolmogorov–Smirnov test and Q-Q plot. The paired t-test, the Wilcoxon rank-sum test or Pearson’s chi^2^ test was applied to test for differences between groups. Intraclass correlation coefficient (ICC) and Bland–Altman plots were applied to describe the agreement between quantification in EDI and conventional OCT mode. Limits of agreement (LoA) for non-parametric data were estimated by quantile estimation and median was used instead of mean for visualization in Bland–Altman plot [26,27].

## 3. Results

The study population had a mean age of 64.88 ± 9.29 years, 55% were male and 56.70% had macular edema on the day of imaging. Patients with DR and RVO did not differ significantly in age, sex, presence of macular edema, central macular volume and central macular thickness (*p* > 0.05) (Table 1).

Regions of interest (ROI) and measurements within the central 3 mm are shown in Figure 1.

Despite the cystoid space and retinal layers appeared to be less contrasted in the EDI image (Figure 1B), the quantification of all three OCT biomarkers was not significantly different in EDI and conventional OCT mode for the entire cohort (for all biomarkers *p* > 0.05) (Table 2). The intraclass correlation coefficient (ICC) was 0.78 for hyperreflective foci, 0.90 for ellipsoid zone reflectivity ratio and 0.80 for choroidal vascularity index; however, intra- and subretinal fluid can cause signal blockage and therefore challenge the analysis of underlying or surrounding structures; therefore, differences between patients with and without macular edema on the day of imaging were compared. In eyes with macular edema (34 from 60 patients), there was a significant difference in the mean number of counted hyperreflective foci between EDI and conventional OCT (conventional: 80.00 ± 33.70, with EDI: 92.08 ± 38.11, mean difference: 12.09, *p* < 0.05). Determination of ellipsoid zone reflectivity ratio and choroidal vascularity index with or without EDI mode was not significantly different in eyes with macular edema (*p* > 0.05). In eyes without macular edema, the quantification of all three biomarkers was similar (*p* > 0.05). Regardless of the presence of macular edema, quantification of ellipsoid zone and choroidal vascularity index showed a mean difference close to zero, while the mean difference of counted hyperreflective foci was 12.09 in eyes with ME and 1.19 in eyes without ME. Overall, for the entire cohort and all subgroups, ICC ranged from 0.76 to 0.91, which indicated a good agreement.

**Figure 2:**
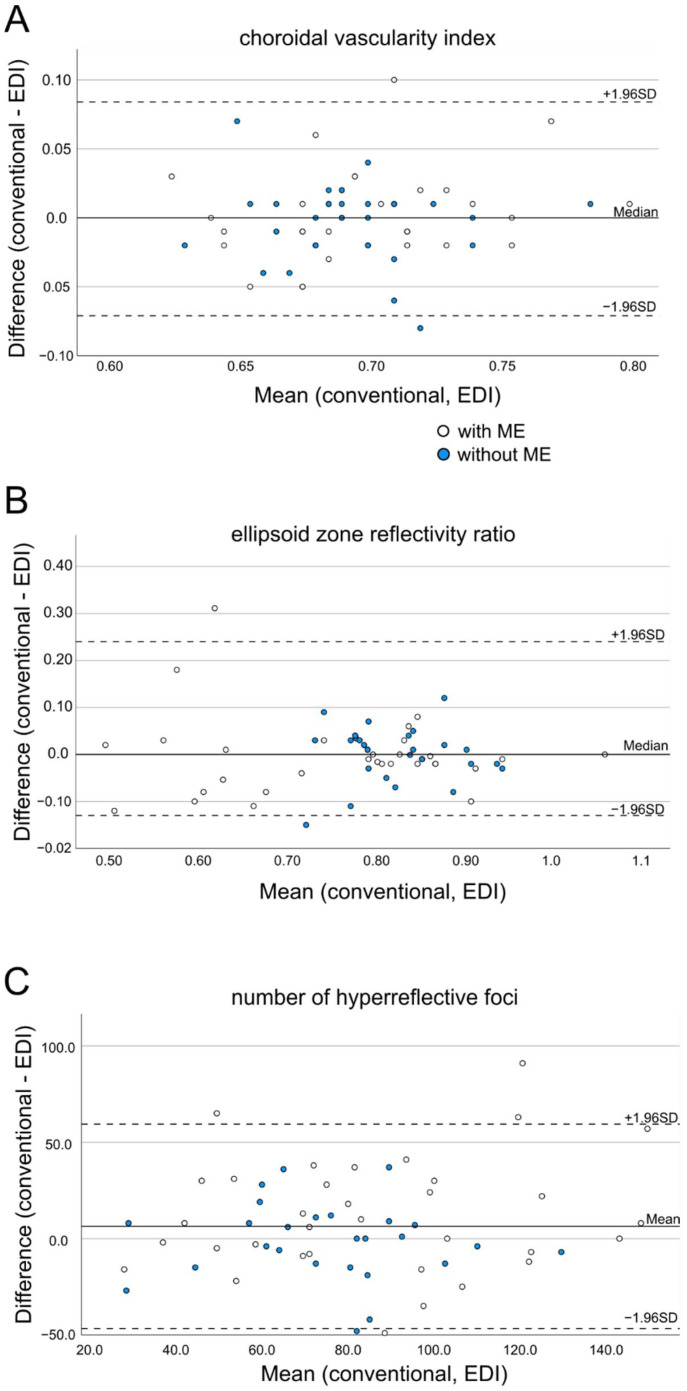
Bland–Altman plots show the differences of quantification of hyperreflective foci (**A**), ellipsoid zone reflectivity ratio (**B**) and choroidal vascularity index (**C**) in OCT scans with and without EDI mode. (**A**–**C**) White and blue dots represent patients with macular edema and without macular edema on the day of imaging. Dashed lines indicate upper and lower limits of agreement (LoA), black line represents mean or median.

Agreement between quantification with and without EDI mode in SD-OCT was visualized in Bland–Altman plots for all three biomarkers (Figure 2). For choroidal vascularity index and ellipsoid zone reflectivity ratio, 3.3% of all patients fell outside the 95% limits of agreement (LoA) (*n* = 2, for each parameter: one DR and one RVO patient). For hyperreflective foci, five patients fell outside the 95% LoA (three DR and two RVO patients).

## 4. Discussion

We compared the automated quantification of three retinal and choroidal OCT biomarkers that are frequently used in SD-OCT with and without EDI mode. We focused on the agreement between SD-OCT with and without EDI mode and evaluated the effect of macular edema on automated quantification.

The value of OCT biomarkers for treatment response and visual outcome prediction has been intensively investigated in recent years. The search for further useful biomarkers in treatment surveillance is driven by the unmet need for personalized treatment that appropriately responds to the heterogeneity of individual patient profiles. Aside from clinical parameters such as the visual acuity (VA) at baseline and the frequency of injections, which are major predicting factors for visual outcome [28,29,30,31], some structural OCT biomarkers have been proposed as predictors for treatment outcome as well. In the literature, the correlation of baseline VA and visual outcome at 1 year ranged from 22 to 46%, thus indicating that there might be other parameters involved in the explanation of visual acuity changes during anti-VEGF therapy [32,33]. Several investigators reported an association of ellipsoid zone integrity with VA changes of up to five years after treatment initiation in diabetic macular edema [11,31] and its predicting role for VA changes during the first year of treatment in neovascular age-related macular degeneration [34]. Ciulla et al. conducted a post hoc analysis of six clinical trials, including 1063 patients with retinal vascular diseases and concluded that EZ integrity accounts for 26.4% and central-subfield thickness for 21.2% of the total variation in visual acuity [30]. It seems that one single parameter might not fully explain visual acuity changes during treatment. Hence, the combination of several OCT biomarkers poses an interesting approach to provide context to clinical decision making and VA prediction. Aside from ellipsoid zone integrity, both hyperreflective foci and choroidal vascularity index have been discussed as predictive factors for treatment response in diabetic macular edema and retinal vein occlusion [8,17,35]; however, evaluating several OCT biomarkers on the same OCT B-scan requires robust imaging quality in all retinal and choroidal layers. Verner-Cole et al. evaluated the visibility of the vitreoretinal surface and subfoveal choroidal thickness in 1050 nm and 870 nm SD-OCT scans with and without EDI mode. They concluded that the manual measurement of subfoveal choroidal thickness in 870 nm SD-OCT with EDI mode was non-inferior to 1050 nm SD-OCT scans. The 1050 nm SD-OCT with EDI mode yielded the best agreement among raters for choroidal thickness, but visualization of the choroid was slightly improved at the expense of visibility of vitreoretinal interface [36]; therefore, for comprehensive analysis of both retinal and choroidal biomarkers, using 870 nm SD-OCT B-scans in EDI mode, as applied in our study, appeared to be suitable to provide adequate image resolution from the inner retinal layers to the choroid.

Our study demonstrated that automated quantification of different OCT parameters across retinal and choroidal layers is feasible in one single SD-OCT B-scan in EDI mode. The strength of this approach lies in the semi-automated analysis of both retinal and choroidal OCT biomarkers, the inclusion of only one eye per patient to reduce statistical variance and the evaluation of the impact of macular edema on the automated quantification.

Notably, the mean difference of counted hyperreflective foci in EDI and conventional OCT images was significantly different in patients with macular edema. It seems that the impact of signal alteration by intraretinal fluid for quantification in EDI and non-EDI images was more pronounced on the quantification of OCT biomarkers that include the inner retinal layers such as the numbers of HF than those in the outer retinal layers or choroid, such as the EZR and the CVI; however, to confirm this observation, we should include and evaluate other OCT biomarkers of the inner retinal layers we had deliberately omitted from the study, such as disorganization of inner retinal layers (DRIL). Applying variation to the existing protocol for HF quantification could potentially mitigate the differences, but we chose to adhere to the original protocols as these were validated by the authors. In the literature, reports about the impact of intraretinal fluid on signal alteration in OCT are limited. For example, Hosseini et al. reported that macular edema decreased signal strength of macular OCT scans more than in retinal nerve fiber layer scans [37]. So far, the impact of macular fluid on automated quantification of OCT biomarkers remains poorly understood. As this study focused on comparing EDI and conventional OCT at a single time point, we did not include follow-up images to assess the temporal change of quantified HF. In order to improve comparability, it would be preferable to follow patients using the same OCT device platform and imaging mode; therefore, despite initially different numbers of counted HF in EDI and non-EDI mode, if subsequent images are applied with the same platform and provided that the changes of quantified HF are the same within each imaging modality, the difference between EDI and non-EDI mode assumably will not impact OCT biomarker assessment. Overall, further research is needed to study the relationship between macular edema and the quantification of OCT biomarkers, as retinal fluid fluctuations are common for patients with retinal vascular diseases requiring anti-VEGF therapy. We quantified hyperreflective foci from the retinal nerve fiber layer to the external limiting membrane, thus including both inner and outer retinal layers. It would be interesting to evaluate other potential differences in the automated quantification of HF in different retinal layers and the effect of the volume and location of macular edema. We note, for example, that Huang et al. reported that HF in the outer retinal layers correlated more with enhanced 1-year visual outcome in diabetic macular edema than HF in the inner retinal layers [35].

The main limitations of our study are the relatively small cohort size, a single time-point of imaging, the need for the manual selection of the region of interest, which can induce bias and we made no comparison to other SD-OCT devices. Furthermore, we have not compared our results to SS-OCT. Imaging with 1050 nm wavelength would have an additional advantage of nearly zero-dispersion in water, resulting in subsequent enhancement of image resolution and sensitivity [38]; however, various studies have demonstrated comparable results in manual quantification analysis of OCT biomarkers such as choroidal thickness between SD- and SS-OCT [22,39]. In addition, the protocol for choroidal vascularity index that was used in this study was derived from Agrawal et al., who also used an SD-OCT device for quantification [15]. Hoseini-Yazdi et al. demonstrated that an averaging of 30 SD-OCT B-scans in EDI mode provided accurate manual measures of choroidal thickness in young, healthy eyes [40]. In our study, all images were averaged from 50 B-scans and provided a good signal-to-noise ratio.

A possible advantage of EDI mode over conventional OCT scans is the better visibility of the posterior choroidal borders, which facilitates the selection of the ROI [36]. This improved visibility is of particular importance for patients with thickened choroids. In our study, all ROI were outlined in the EDI image, and the choroidal posterior border was clearly visible in all images. We transferred the ROI mask from the EDI image to the conventional OCT B-scan; therefore, we did not compare the visibility of the posterior border between the EDI and conventional OCT B-scans.

Aside from morphological alterations, changes in the optical reflectivity of different retinal layers and RPE could be excellent biomarkers for various retinal diseases as well. Biomarkers based on optical reflectivity, such as EZR or the optical reflectivity of the inner retinal layer, which has been shown to be altered in acute retinal ischemia, have the advantage that alteration can be detected before the appearance of morphological changes [41,42,43]. Overall, a better understanding of the scatter properties and reflectivity profiles of retinal layers in different OCT modes will contribute to the interpretation of OCT biomarkers in retinal diseases.

In conclusion, this study demonstrated excellent reproducibility of retinal and choroidal OCT biomarkers, namely ellipsoid zone reflectivity ratio and choroidal vascularity index, in SD-OCT with and without EDI mode regardless of the presence of a macular edema. Hence, quantification of retinal and choroidal biomarkers can be derived from one single 870 nm SD-OCT B-scan in EDI mode.

## Figures and Tables

**Table 1:** Demographics and characteristics for patients with diabetic retinopathy (DR) (*n* = 27) or retinal vein occlusion (RVO) (*n* = 33).

		Overall (*n* = 60)		DR (*n* = 27)		RVO (*n* = 33)		
Baseline Variables		Mean (SD)	Median (Q1; Q3)	Mean(SD)	Median (Q1; Q3)	Mean (SD)	Median (Q1; Q3)	*p*
Age (years)		64.88 (9.29)	65.00 (59.25; 72.25)	62.51 (9.68)	64.00 (60.00; 67.00)	66.81 (8.62)	66.00 (59.00; 74.50)	0.07 °
With EDI	frames	48.97 (7.35)	50.00 (49.00; 51.00)	48.70 (7.70)	50.00 (49.00; 51.00)	49.18 (7.12)	50.00 (49.00; 51.00)	0.80 °
	SNR	31.13 (3.60)	31.00 (29.00; 33.00)	30.70 (3.67)	31.00 (29.00; 33.00)	31.48 (3.56)	32 (29.00; 33.50)	0.40 °
Without EDI	frames	48.68 (7.19)	50.00 (49.00; 50.75)	48.92 (7.66)	50.00 (50.00; 51.00)	48.48 (6.89)	50 (49.00; 50.00)	0.82 °
	SNR	31.23 (4.43)	31.00 (28.25; 34.00)	31.44 (4,57)	30.00 (29.00; 34.00)	31.06 (4.37)	32 (28.00; 34.50)	0.74 °
CMT (µm)		310.28 (98.99)	289.50 (252.25; 332.25)	285.00 (70.11)	286.00 (239.00; 313.00)	330.96 (114.43)	296 (259.00; 358.00)	0.07 °
CMV (mm^3^)		0.24 (0.078)	0.23 (0.20; 0.26)	0.22 (0.05)	0.22 (0.19; 0.25)	0.26 (0.09)	0.23 (0.20; 0.29)	0.07 °
		*n* (%)		*n* (%)		*n* (%)		
Male sex		33 (55.00)		15 (55.60)		18 (54.50)		0.93 *
Study eye OD		28 (46.70)		12 (44.40)		16 (48.50)		0.75 *
Macular edema present		34 (56.70)		14 (51.90)		20 (60.60)		0.49 *

SNR = signal to noise ratio; EDI = enhanced depth imaging mode; CMT = central macular thickness; CMV = central macular volume: OD = right eye. SD = standard deviation; Q1 = first quartile; Q3 = third quartile. *p* values were calculated with ° paired Student’s *t*-test or * Pearson’s chi^2^ test.

**Table 2:** Comparison between SD-OCT without and with EDI mode in OCT biomarkers measurements.

OCT Biomarker	Group	Without EDI (Mean ± SD)	With EDI (Mean ± SD)	Mean Difference	*p*-Value *	ICC (95% CI)
Hyperreflective foci (In number)	Overall	78.30 ± 30.29	84.63 ± 33.71	6.30	0.07 °	0.78 (0.62–0.87)
	With ME	80.00 ± 33.70	92.08 ± 38.11	12.09	0.03 °	0.76 (0.52–0.88)
	Without ME	76.07 ± 25.56	74.89 ± 24.30	−1.19	0.76 °	0.80 (0.56–0.91)
Ellipsoid zone reflectivity ratio (arbitrary unit)	Overall	0.78 ± 0.12	0.78 ± 0.12	0.00	0.60 *	0.90 (0.84–0.94)
	With ME	0.76 ± 0.14	0.75 ± 0.15	0.00	0.31 °	0.91 (0.83–0.96)
	Without ME	0.82 ± 0.07	0.83 ± 0.06	0.00	0.69 °	0.77 (0.47–0.89)
Choroidal vascularity index (arbitrary unit)	Overall	0.69 ± 0.03	0.69 ± 0.04	0.00	0.91 *	0.80 (0.67–0.88)
	With ME	0.70 ± 0.37	0.70 ± 0.45	0.00	0.39 °	0.83 (0.66–0.91)
	Without ME	0.70 ± 0.04	0.69 ± 0.03	0.00	0.50 °	0.77 (0.48–0.90)

ME = macular edema, SD = standard deviation, ICC = intraclass correlation coefficient, CI = confidence interval; ° Paired Student’s *t*-test, * Wilcoxon rank-sum test.

## Data Availability

All available data generated or analyzed during this study are included in this published article.
